# Improved Detection of Lung Fluid With Standardized Acoustic Stimulation of the Chest

**DOI:** 10.1109/JTEHM.2018.2863366

**Published:** 2018-08-21

**Authors:** Adam Rao, Simon Chu, Neil Batlivala, Samuel Zetumer, Shuvo Roy

**Affiliations:** Department of Bioengineering and Therapeutic SciencesUniversity of California at San FranciscoSan FranciscoCA94158USA; School of MedicineUniversity of California at San FranciscoSan FranciscoCA94143USA; Consultant

**Keywords:** Acoustic sensors, actuators, biomedical acoustics, transfer function, classification algorithms

## Abstract

Accumulation of excess air and water in the lungs leads to breakdown of respiratory function and is a common cause of patient hospitalization. Compact and non-invasive methods to detect the changes in lung fluid accumulation can allow physicians to assess patients’ respiratory conditions. In this paper, an acoustic transducer and a digital stethoscope system are proposed as a targeted solution for this clinical need. Alterations in the structure of the lungs lead to measurable changes which can be used to assess lung pathology. We standardize this procedure by sending a controlled signal through the lungs of six healthy subjects and six patients with lung disease. We extract mel-frequency cepstral coefficients and spectroid audio features, commonly used in classification for music retrieval, to characterize subjects as healthy or diseased. Using the $K$-nearest neighbors algorithm, we demonstrate 91.7% accuracy in distinguishing between healthy subjects and patients with lung pathology.

## Graphical abstract

An acoustic transducer and digital stethoscope system are proposed as a targeted solution for detection of fluid in the lung. In a clinical trial, the system demonstrated 91.7% accuracy in distinguishing between healthy subjects and subjects with lung pathology.

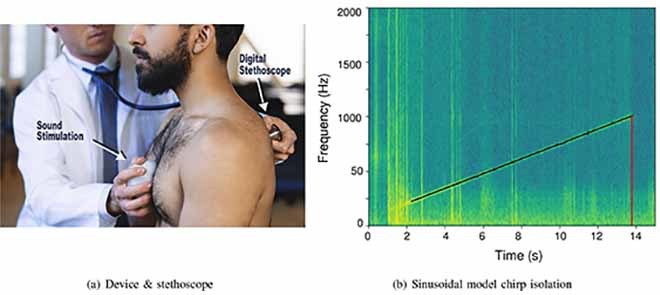

## I. Introduction

Respiratory diseases are a leading cause of death worldwide [1]. Shortness of breath, or dyspnea, is a common chief complaint among patients presenting to the hospital, accounting for 3.7 million visits to emergency departments in the US in 2011 [2]. Rapid and reliable diagnosis of the undifferentiated adult patient presenting with acute dyspnea is critical for appropriate triage, medical management, and identification and prevention of imminent respiratory collapse. Physical examination is the standard procedure for initial evaluation of patients who present with respiratory symptoms. A respiratory physical exam consists of several procedures, including listening to the patient’s thorax with a stethoscope (auscultation) and tapping to check for areas of dullness corresponding to pathology (percussion) [3].

Percussion is a technique in which one introduces a sound stimulus through the chest wall and detects a change in quality associated with the presence of lung pathology. By tapping on the patient’s back and listening for certain sounds, a clinician can determine if a lung field is abnormally occupied by air, fluid, or solid mass. Structural changes induced by disease cause alterations in acoustic transmission of frequencies through the thoracic cavity [4]. While valuable, appropriate execution of this technique and interpretation of the findings are both subjective and highly skill dependent [5]. Exam results often suffer from a high degree of variability and a low interobserver agreement [6]. This qualitative physical exam has led to the introduction of adjunctive imaging modalities to assist in the diagnosis of respiratory disease.

Radiographic imaging is one of the most common diagnostic methods used to evaluate the presence of lung disease that might not be detected by the physical exam. While a key supplement in the diagnosis and management of respiratory patients, the chest radiograph has its drawbacks. The use of high-energy ionizing radiation to penetrate tissue for imaging can lead to mutations that increase the risk of cancer. In addition, certain obstructive airway diseases such as chronic obstructive pulmonary disease (COPD) and asthma may also be missed in an x-ray analysis [7]. Furthermore, the equipment may be cost-prohibitive in resource-poor settings, where the burden of respiratory disease is greatest. For example, the cost of deploying an x-ray machine in Northern India is estimated at $51,500 plus $5,900 per year to operate [8]. Consequently, adjunctive pulmonary monitoring methods that are low-risk, inexpensive, and accurate are the subject of active research.

Ultrasound does not carry radiation risks and has established diagnostic utility for the heart, kidneys and other major organs. Unfortunately, its utility for directly imaging lung tissue is still being researched [9]. This is largely due to acoustic impedance mismatch at high frequencies (on the order of MHz) between the chest wall and air inside the lungs, resulting in reflection of ultrasound waves at the surface of the lungs [5]. Lung ultrasound, like the physical exam, also has limitations that are both operator- and patient-dependent. Utilizing ultrasound and correctly interpreting its findings requires extensive formal training.

Here, we present a proof of concept for the acoustic detection of structural lung pathologies, utilizing a low-cost acoustic transducer to provide a fixed signal input and a digital stethoscope system paired with a novel audio processing algorithm for automated classification. Standardizing the acoustic stimulation is designed to reduce patient and operator variability. The cost for an acoustic based system is hundreds rather than thousands of dollars, providing a feasible and scalable diagnostic solution for the developing world. Our investigation into distinguishing features characteristic of normal and abnormal lung function provides a foundation upon which future studies that examine disease-specific lung changes can be based. This system aims to provide rapid and accurate diagnosis in the emergent patient who presents in respiratory distress.

## II. Background

### A. Related Work

The development of computerized lung sound analysis has led to several studies investigating classification of breath sounds as healthy or pathological [10]–[14]. Lung sounds are heard over the chest during inspiration and expiration. They are non-stationary and non-linear signals, requiring a combined time and frequency approach for accurate analysis [15]. Processing typically involves recording the breath sounds with an acoustic sensor, extracting audio features from the recordings, and feeding these features into a classifier. Lung sounds are typically recorded using contact microphones, such as an electronic stethoscope. Classification features are commonly based on autoregressive (AR) modelling, mel-frequency cepstral coefficients (MFCC), spectral energy, and the wavelet transform [16]. For classification, artificial neural network (ANN) and K-nearest neighbors (KNN) are commonly used. Previous work utilizing KNN and ANN classification to distinguish between healthy and pathological lung sounds reported values ranging from 69.7–92.4% for accurate classification [12], [14], [17], [18]. Although the usage of breath sound analysis shows potential for accurate classification, the large range in accuracy reported in prior work motivates the need for a standardized approach. Changes observed in recorded breath sounds could be a result of both the differences in structure of the system or the result of intersubject and intrasubject variability between breath cycles.

Compared to lung sound analysis, the study of how fixed external sounds travel through the lungs offers much room for development. A 2014 study reported sending a chirp in the range of 50–400 Hz into the chest using a transducer, and demonstrated measurable changes in sound transmission for air accumulation in the chest [5]. Previous work on this device investigated changes in sound transmission during lung fluid accumulation due to pneumonia, sending a chirp into the chest in the range of 50–500 Hz using a surface exciter transducer [19]. The present study expands the frequency range from 500 Hz to 1000 Hz, as our signal-to-noise ratio in this range is sufficiently high for chirp detection and analysis. To our knowledge, no work has yet been done in classifying patients with pulmonary pathology based on a fixed percussive input acoustic signal.

### B. Approach and Statement of Contributions

The objective of this paper is to present an alternative to automated breath sound analysis that provides improved performance for lung pathology assessment. The key idea is to provide a fixed input signal to the chest, which sweeps across the frequencies of interest for analysis. By using a fixed input signal, we ensure that differences observed are purely a result of differences in the structure of the system being probed. This allows calculation of the acoustic frequency response of the chest and extraction of relevant features for classification.

The contributions of the paper are threefold. First, we present a noise-robust method of chirp signal tracking for acoustic system identification. Second, we provide a proposed set of classification features for healthy compared to pathological lungs, using both acoustic and clinical features for analysis. Finally, we present our classification results for the device and compare them to existing methods of breath sound analysis, comparing 216 recordings from six healthy subjects to 216 recordings from six patients. By using acoustic features to distinguish between these two groups, we achieved a classification accuracy of 92%.

## III. Methods

### A. Study Design and Subjects

The study was conducted at University of California, San Francisco Medical Center under approved institutional review board study number 15-16814. The study cohort consisted of patients with respiratory disease and healthy controls. The group of patients with respiratory disease consisted of English-speaking patients between 18–85 years of age who presented to the emergency department with a chief complaint of acute shortness of breath (dyspnea). Our study includes patients presenting with decompensated heart failure, chronic obstructive pulmonary disease (COPD), and asthma, the most common diagnoses among patients presenting to Emergency Department with a complaint of acute dypsnea and signs of respiratory distress [20]. The diagnosis of respiratory disease was provided by the treating physician, and was obtained from the medical chart for comparison. The healthy controls group consisted of individuals who did not have active respiratory symptoms or a medical history of confounding pulmonary pathology at the time of enrollment. All participants provided written informed consent prior to inclusion in the study.

### B. Instrumentation

**FIGURE 1.**
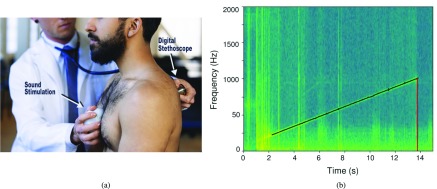
(a) The device is placed on the patient’s sternum to provide a fixed input chirp sweeping from 50 to 1000Hz into the chest. This signal is recorded using the Eko digital stethoscope for chirp isolation and analysis. (b) Sinusoidal modeling results in improved chip isolation when compared with the standard onset detection. The model tracks the chirp and returns the value at 1000 Hz when the chirp terminates, shown here occurring at 13.8 seconds. (a) Device & stethoscope. (b) Sinusoidal model chirp isolation.

We used a hand-held acoustic device to emit a controlled audio signal into the chest, shown in Figure 1(a) [19]. The device consists of a lithium polymer battery, a printed circuit board (PCB), and surface exciter, all contained in a 3D-printed plastic enclosure. The signal is a chirp of uniform intensity that increases linearly from 50 Hz to 1000 Hz over a 14 second period. A ‘chirp’ signal was chosen to provide adequate power in each frequency band for analysis, and was based on preliminary studies with the device [19], [21]. For recording, we used the *Eko Core* digital stethoscope, which demonstrated a sufficiently flat frequency response in the frequency range of our analysis (50Hz–2000Hz) [22]. Since a fixed signal was used for both healthy subjects and patients, the transfer functions of the transducer and microphone were constant and did not contribute to differences between the two groups.

### C. Frequency Range Selection

COPD and asthma are highly prevalent diseases; both are associated with airway inflammation and obstruction, leading to air trapped in the lungs [23]. For asthma, the median frequency of lung sound changes has been reported in the range of 240 Hz, while lung sound changes for COPD have been reported under 400 Hz [16], [24]. Heart failure and pleural effusion result in an analogous build-up of fluid in the lung tissue (parenchyma) and surrounding cavity due to leakage from the circulatory system. Diseases that result in trapped air and fluid lead to alterations in sound transmission due to the differences in acoustic characteristics of different media [25], [26]. The dominant frequency of parenchymal pathology ranges from 200 to 2000 Hz. Previous work investigating sound transmission in the chest cavity demonstrated significant attenuation at frequencies above 1000 Hz, suggesting an area of interest for analysis below this cutoff [25], [27], [28]. These frequency considerations led to the choice of stimulation frequencies between 50 to 1000 Hz.

### D. Recording Procedure

The clinical study visit consisted of two types of audio recordings through the *Eko Core* digital stethoscope: percussion sounds and breath sounds. Recordings were obtained at six different locations: the right and left upper, middle, and lower lung fields. The recordings were taken from symmetric locations from the apices to the lung bases according to Bates’ guide for physical examination [3]. Percussion sounds and breath sounds were each recorded in triplicate, leading to 36 recordings for each patient, each 15 seconds long. Percussion sounds were produced by the acoustic device, which emitted a controlled chirp held against the body of the sternum. Breath sounds consisted of recordings of the patient’s respiration without the device. In additional to audio recordings, thoracic circumference and demographic data including age, gender, height, weight were collected for each patient.

### E. Audio Pre-Processing

Several pre-processing steps were performed to isolate the signal of interest. Each stethoscope recording was 15 seconds long; since the chirp lasted for 14 seconds, it was surrounded by periods of silence and low-energy background noise. Areas of silence are discarded when computing the features of the audio because certain features which rely on spectral energy would be adversely affected by their inclusion. To identify and crop the recordings to isolate the chirp, we used a sinusoidal model with sine tracking.

Sinusoidal analysis approximates a signal with a sum of a finite number of sinusoidal with time-varying amplitude and frequency [29]. An input sound s(t) is modeled by

\begin{equation*} s(t) = \sum _{r=1}^{R}{A_{r}(t)cos[\theta _{r}(t)] }\end{equation*}

where $A_{r}(t)$ and $\theta _{r}(t)$ are the instantaneous amplitude and phase function of the sinusoid, respectively. This model assumes that the sinusoids are stable partials of the sound and that each has a slowly changing amplitude and frequency. The sinusoidal model has several parameters that can be adjusted according to the sound being analyzed. Similar to the short-time Fourier transform (STFT), the sinusoidal model performs successive discrete Fourier transforms (DFT) by utilizing a moving windowing function.

Parameters were chosen specific to our stimulation signal. The size of our DFT was 2048 points, chosen to provide adequate frequency resolution for our sampling rate of 4 kHz. We chose the Blackman-Harris window because its main lobe includes most of the energy, which reduces the artifacts of windowing. For a chirp signal, the frequency continuously changes, which necessitates a shorter window. Thus, we chose a window size of 401 samples with additional points set to zero with zero padding. This window was moved by a hop size of 50 samples to avoid artifacts that arise when the hop size is too large. For each frame of the STFT, the sinusoidal analysis identified the largest sinusoidal component. This sinusoid was tracked for the duration of the signal, and the time and frequency tuples were used to isolate the chirp, as seen in Figure 1(b). These parameter choices allowed the chirp to be isolated despite background noise and resulted in superior performance compared to standard methods of acoustic event detection which rely on energy changes between frames [30].

As a final pre-processing step, we applied a cross-fade (a 500 ms fade in and fade out), a technique used when the periodic timing of a signal is difficult to predict, in order to increase the fidelity of the DFT [31].

### F. Frequency Principle Component Analysis

A three-component principal component analysis (PCA) was conducted on the spectra of chirp audio to determine which frequencies contributed most significantly to the variability between subject recordings. We used the FFT to find the power in the chirp within the range of 50 to 1000 Hz. We recorded the variability captured by each of the three principal components and examined which frequencies contributed most to these components. This analysis was completed at a resolution of 8 Hz. Our results were then compared to prior literature to determine if these frequencies corresponded to differences between healthy and diseased subjects.

### G. Audio Features for Classification

From the processed data, we extracted specific features: the Mel-frequency cepstral coefficients (MFCCs) and the spectral centroid of the recordings. MFCCs are a set of coefficients that are commonly used in audio analysis to represent the frequency spectrum of a signal in a compact form [32]. The Mel scale approximates the human auditory system, giving higher weight to differences at lower frequencies. Since we are focusing on frequencies below 1000 Hz, this weighting is suitable for our analysis.

The spectral centroid is another commonly used audio feature that characterizes a spectrum by finding its center of mass [33]. It measures which frequencies are the most prominent across a signal by considering the spectrum as a distribution in which the values are the frequencies and the probabilities are the normalized amplitude. The spectral centroid provides a value for each frame of the signal; these values were then averaged to obtain the average spectral centroid value for the entire signal. The formula for calculation is shown below, where $k$ is the frequency of the signal, $l$ is the frame of the audio signal, $X_{l}(k)$ is the value of the DFT at point k for frame l, and N is the size of the DFT [33].

\begin{equation*} \text {Spectral Centroid = } \frac {\sum _{k=0}^{N/2}{k \cdot X_{l}(k)}}{\sum _{k=0}^{N/2}{X_{l}(k)}}\end{equation*}

For each patient, we averaged the MFCC and spectral centroid values across three trials and six lung regions. An average was obtained because the lung pathologies tend to be diffuse processes affecting multiple lobes of the lung. The MFCC coefficents and spectral centroid value resulted in a set of 11 features to compare for each patient. The open source library Essentia was used to implement both the MFCC calculation and centroid calculation in Python [34].

### H. Algorithmic Classification of Healthy or Diseased Lungs

**FIGURE 2.**
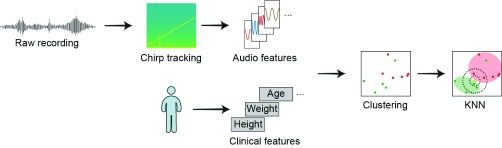
Block diagram of the data analysis process. Sounds were pre-processed using sinusoidal analysis to isolate the chirp. The audio was processed into acoustic features; other clinical features such as height and weight were recorded as clinical features. These features are plotted in the feature space to identify clusters. The feature vectors of sounds from the normal subjects are shown in green, while patient sounds are shown in red. Clustering of two example features (MFCC3 and MFCC8) from the total of 11 features are shown. The 11 features were analyzed using KNN to classify the sounds.

We combined the audio features and patient features into a single feature set in order to classify the subject into the healthy or unhealthy classes; this process is illustrated in Figure 2. We used vector quantization to map our 11-dimensional vector to our 2-dimensional classification vector space [35].

We considered several vector quantization techniques including KNN, support vector machines (SVM), and gaussian mixture models (GMM) [36]. Although neural networks have been shown to produce a very high accuracy classification model for lung sounds [37], due to the size of our data set, the use of a neural network would most likely lead to over-fitting. The KNN algorithm has been demonstrated to be superior to SVM for breath sound (BS) analysis [16]. We performed a comparison of the three classification techniques using the percussion sounds (PS) generated by the device. Our comparison of KNN, SVM and GMM also demonstrated superior accuracy for the KNN. These results together with KNN’s improved interpretability versus GMM, led us to use KNN for our analysis.

The KNN algorithm was used to classify the feature set for each patient as either healthy or unhealthy. KNN classifies participant lung health by choosing the majority class of the K most similar patients. In this case, similarity is determined by the euclidean distance of audio and clinical features between patients. The K value refers to the number of ‘neighbors’ to query when assigning a point to a class. For the analysis, K values were compared across 1, 3, 5, and 7. Since the algorithm takes a majority vote of the closest neighbors to assign a class, even values of K are not used to prevent a tie during the vote. A value of 1 indicates that any point is assigned the class of its closest neighbor, although this value often leads to 100% clustering, it is susceptible to over-fitting. Higher accuracy at lower K values can be interpreted as more densely correlated classes; higher values of K can help account for more noise in the data as more data points are considered to assign a class.

## IV. Results & Discussion

We obtained acoustic recordings from six healthy subjects and six patients with lung disease. The clinical features from these subjects is displayed in Table 1. We first performed a three-component PCA analysis on the 50–1000Hz range of the spectra of chirp recordings. The three principle components accounted for 60, 12, and 5 percent of the variation among spectra. The 830–860Hz range contributed most significantly to the first component, while the 230–260Hz range were the most significant frequencies in the second component. We used MFCC audio features, spectroid audio features, and patient clinical features processed with the KNN clustering algorithm to classify healthy and diseased lung states. The performance of recordings from the device, termed percussion sounds (PS), is evaluated with and without clinical features as shown in Table 2. In addition, PS are compared to standard breath sounds (BS) to evaluate their classification utility, as shown in Table 3. GMM was performed using a full covariance matrix and achieved a classification accuracy of 50.0%; SVM was performed with both a linear and quadratic kernel, yielding a classification accuracy of 75.0%. By comparison, using KNN at its optimum tuning setting, $K$ = 3, we were able to achieve a classification accuracy of 91.7%.

**TABLE 1:** Clinical Features of Healthy Subjects and Patients

Features	Healthy Subjects	Patients
Age	27 ± 5%	54 ± 12%
Gender	50% Male	50% Male
Height (cm)	166 ± 23	166 ± 8
Weight (kg)	72 ± 21	84 ± 20
Thorax Circ (cm)	95 ± 12	106 ± 10

Clinical features used to further refine analysis. Age, Gender, Height, Weight, Thorax Circumference were obtained from each subject.

**TABLE 2:** Comparison of KNN With and Without Clinical Features

Features	K = 1	K = 3	K = 5	K = 7
PS	100%	91.7%	66.7%	66.7%
PS + Clinical	100%	91.7%	91.7%	66.7%

The percussion recordings using the device, percussion sounds (PS) are evaluated with and without clinical features. The clinical features improve performance for the higher K values of 3.

**TABLE 3:** Comparison of KNN Between Percussion Sounds and Breath Sounds Feature Set (With Clinical Features)

Features	K = 1	K = 3	K = 5	K = 7
PS	100%	91.7%	91.7%	66.7%
BS	100%	75.0%	66.7%	66.7%
PS+BS	100%	75.0%	75.0%	58.3%

The percussion recordings using the device, percussion sounds (PS) are compared to the recordings of breath sounds (BS) from the patient. PS sounds can be seen to provide higher classification accuracy than BS.

Our preliminary frequency analysis indicated that the frequency of greatest variability occurred in the 830–860 Hz range as well as in the 230–260 Hz range. The 230–260 Hz range variability lines up with previously reported frequency changes in asthma of 240 Hz [24]. The high level of variability at the 830–860 Hz range could be explained by changes in parenchymal fluid in heart failure and pleural effusion reported to be in the range of 200 to 2000 Hz [16]. However, due to this wide range, further investigation is needed to discriminate the frequency differences for specific disease states. This PCA result suggests that the presence of structural lung disease leads to detectable alterations in the sound transmission across the chest. Additional spectral characteristics such as MFCC and spectral centroid provide more generalizable information about the changes in acoustic transmission.

The accuracy of lung health classification from the controlled audio signal was tested across 12 patients and a variety of values of $K$ for the KNN algorithm. Our highest classification accuracy was 91.7%, obtained by using the features from our acoustic device and the clinical features of each patient. Using our classifier on the recordings resulted in just a single misclassification out of 12 subjects.

From Table 2, comparing the KNN classification accuracy obtained from just PS features and PS features combined with clinical features, we can see that the inclusion of clinical features improved accuracy when $K$ = 5 from 66.7% to 91.7%. This aligns with the notion that these clinical features provide valuable information when classifying signal transmission. Sound transmission through the thorax is affected by the size of the chest which contributes to variability between subjects; by including thorax circumference and other clinical features we improve our classification accuracy.

From Table 3, we can see that classification using PS features outperformed classification based on BS features when $K$ = 3 and $K$ = 5. Not only did the PS features perform better, the introduction of BS features with PS features hindered classification accuracy when $K$ = 5 and 7 compared to the PS features alone. Providing a controlled audio input across patients provides a cleaner signal than breath sounds which vary between patients and between trials. Furthermore, inclusion of BS appears to add noise to the system, reducing the accuracy of the KNN algorithm. The improved performance therefore motivates the use of a standardized audio signal rather than breath sounds for acoustic analysis of lung fluid.

There are limitations to this analysis; the recordings were collected from 12 subjects, which prevented splitting the data into training and testing sets and increased the risk of over-fitting. Despite this limitation, because the clustering was performed identically for both the PS and BS features, the risk of overfitting would apply equally to both data sets. An additional limitation is the difference in age between the control group and the patient group. Future studies to control for age related lung changes will require a larger patient cohort with age matched controls.

This work also prompts further study of lung disease-specific classification to distinguish between respiratory conditions and to aid in differential diagnosis. Extending our analysis to allow for localized classification could be particularly helpful in conditions such as pneumonia, tuberculosis, or lung cancer, which can affect individual lobes of the lung. Larger disease-specific studies provide an opportunity to improve classification accuracy as different diseases lead to different structural changes. Another area of interest would be to measure changes in audio features over time, as a patient’s clinical course and corresponding fluid buildup or trapped air in the lungs worsens or resolves. This has particularly relevant applications in respiratory conditions such as congestive heart failure, and could allow physicians the ability to monitor lung fluid status, reducing heart failure exacerbation and costly hospital admissions.

## V. Conclusion

In this analysis we demonstrated that the presence of structural lung disease leads to detectable frequency differences using an PCA analysis of the frequencies from an FFT of the recorded signal. KNN clustering of standard MFCC and spectroid audio features extracted from the recordings resulted in successful segmentation of healthy and pathological cases. Furthermore we demonstrated that classification of the standardized stimulation features outperforms classification based on breath sound features. This may be due to the amount of variability in breath sound recordings and is evidence toward one of our initial assumptions: a controlled, patient-independent input signal provides the most robust classification features. Future work will focus on collecting more patient data from specific disease states and applying more flexible classification techniques such as neural networks to extract new and more complex patterns from its features.
